# 8-oxoguanine causes spontaneous *de novo* germline mutations in mice

**DOI:** 10.1038/srep04689

**Published:** 2014-04-15

**Authors:** Mizuki Ohno, Kunihiko Sakumi, Ryutaro Fukumura, Masato Furuichi, Yuki Iwasaki, Masaaki Hokama, Toshimichi Ikemura, Teruhisa Tsuzuki, Yoichi Gondo, Yusaku Nakabeppu

**Affiliations:** 1 Department of Medical Biophysics and Radiation Biology, Faculty of Medical Sciences, Kyushu University, Fukuoka 812-8582, Japan; 2 Division of Neurofunctional Genomics, Department of Immunobiology and Neuroscience, Medical Institute of Bioregulation, Kyushu University, Fukuoka 812-8582, Japan; 3 Research Center for Nucleotide Pool, Kyushu University, Fukuoka 812-8582, Japan; 4 Mutagenesis and Genomics Team, RIKEN BioResource Center, Tsukuba 305-0074, Japan; 5 Radioisotope Center, Kyushu University, Fukuoka 812-8582, Japan; 6 Department of Computer Bioscience, Nagahama Institute of Bio-Science and Technology, Nagahama 526-0829, Japan; 7 Research Fellow of the Japan Society for the Promotion of Science

## Abstract

Spontaneous germline mutations generate genetic diversity in populations of sexually reproductive organisms, and are thus regarded as a driving force of evolution. However, the cause and mechanism remain unclear. 8-oxoguanine (8-oxoG) is a candidate molecule that causes germline mutations, because it makes DNA more prone to mutation and is constantly generated by reactive oxygen species *in vivo*. We show here that endogenous 8-oxoG caused *de novo* spontaneous and heritable G to T mutations in mice, which occurred at different stages in the germ cell lineage and were distributed throughout the chromosomes. Using exome analyses covering 40.9 Mb of mouse transcribed regions, we found increased frequencies of G to T mutations at a rate of 2 × 10^−7^ mutations/base/generation in offspring of *Mth1/Ogg1/Mutyh* triple knockout (TOY-KO) mice, which accumulate 8-oxoG in the nuclear DNA of gonadal cells. The roles of MTH1, OGG1, and MUTYH are specific for the prevention of 8-oxoG-induced mutation, and 99% of the mutations observed in TOY-KO mice were G to T transversions caused by 8-oxoG; therefore, we concluded that 8-oxoG is a causative molecule for spontaneous and inheritable mutations of the germ lineage cells.

Evolution requires *de novo* germline mutations that are newly generated in germ lineage cells and inheritable to the offspring. It is evident that germline mutations occur, because sporadic and deleterious mutations that cannot be transmitted to offspring continuously appear in human populations^1^,^2^,^3^,^4^. The human *de novo* germline mutation rate is estimated to be 1.20 × 10^−8^/nucleotide/generation^1^. However, the cause and mechanism of mutations in the germ cell lineage remain unclear. We hypothesized that the cause of these mutations would be endogenously and spontaneously generated and remain in the germ cell lineage. 8-oxoG is one of the candidate molecules for causing germline mutation, because it is endogenously generated by reactive oxygen species (ROS) derived from cellular respiration, constitutively exists in DNA^5^ and is known to cause G to T and A to C transversion mutations by the ability to pair with A as well as C during DNA replication^6^,^7^,^8^.

Mammals possess three enzymes to avoid 8-oxoG-induced mutations. MTH1 (*mutT* homologue 1, NUDT1) degrades 8-oxodGTP in the nucleotide pool to prevent its incorporation into DNA^9^. OGG1 (8-oxoG DNA glycosylase) excises 8-oxoG from DNA^10^,^11^, and MUTYH (*mutY* homologue, adenine DNA glycosylase) removes adenine misincorporated opposite 8-oxoG in DNA^12^. We and other groups have reported that mice deficient in these enzymes are prone to developing cancer, indicating a mutator phenotype in somatic cells^13^,^14^,^15^,^16^. MUTYH is also responsible for MUTYH-associated polyposis in humans^17^.

To evaluate the contribution of 8-oxoG to *de novo* germline mutation, we established the *Mth1/Ogg1/Mutyh* triple knockout (TOY-KO) mice, in which unrepaired endogenous 8-oxoG accumulates in the genome DNA. In this paper, using the TOY-KO mice, we showed that 8-oxoG causes G to T mutations in germ lineage cells (Supplementary Fig. S1 online).

## Results

### Spontaneous mutations increased in *Mth1^−/−^/Ogg1^−/−^/Mutyh^−/−^* (TOY-KO) mice

**Figure 1:**
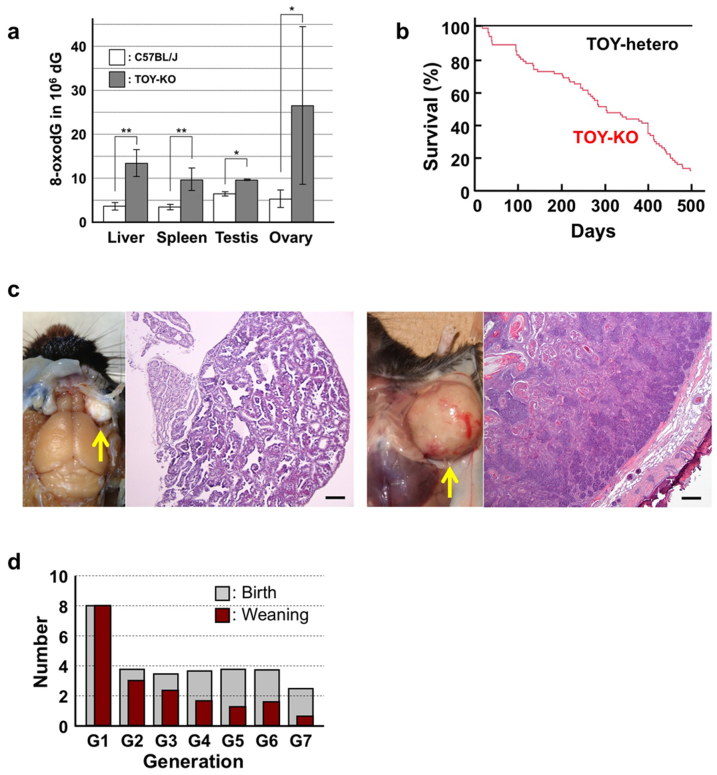
Phenotype of TOY-KO mice. (a) Accumulation of 8-oxodG in TOY-KO mouse tissues. LC-MS/MS was used to determine the amount of 8-oxodG^29^. Data are presented as the means ± SD. Wilcoxon tests were used to analyze differences between TOY-KO (gray) and C57BL/6J:Jcl (open) mouse tissues (* *P* < 0.05; ** *P* < 0.001). (b) Survival of TOY-KO mice. The survival curve of TOY-KO mice (n = 56, indicated in red) was compared with that of *Mth1^+/−^/Ogg1^+/−^/Mutyh^+/−^* (TOY-hetero) mice (n = 14, indicated in black). (c) A Harderian gland tumor (left) and a trichoepithelioma (right) observed in a TOY-KO mice (indicated by arrows). Hematoxylin and eosin staining of each tumor is shown. Scale bars, 200 μm. (d) Numbers of newborn and weaned mice. Gray and red bars indicate the numbers of newborn and weaned mice in each generation of TOY-KO mice, respectively.

**Figure 2:**
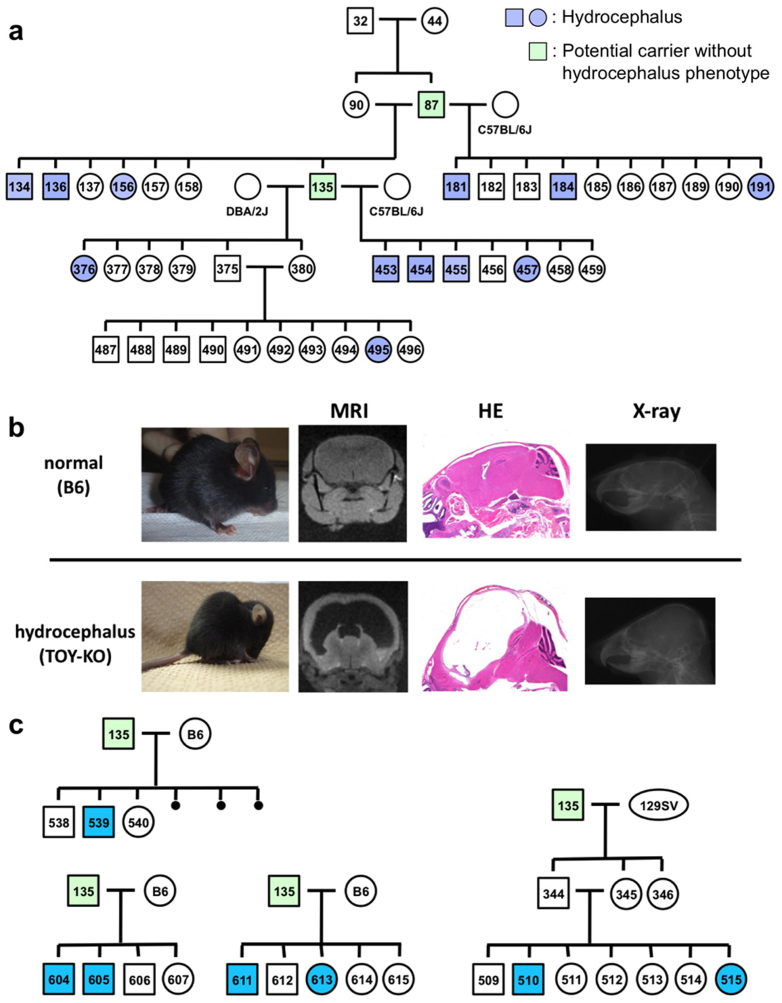
Phenotypic variations observed in the progeny of TOY-KO mice. (a) The hydrocephalus trait was transmitted to the next generation in the TOY-KO pedigree. A hematoxylin/eosin-stained section showing the typical features of the hydrocephalus trait. Blue indicates a mouse with hydrocephalus, and green indicates a mouse carrying the causative mutation without the hydrocephalus phenotype (also shown in Supplementary Fig. S2 online). (b) Hydrocephalus. MRI, hematoxylin/eosin staining and X-ray images of normal (C57BL/6J) and hydrocephalus TOY-KO mice are shown in the upper panel. MRI images were obtained using an MRI mini SA (DS Pharma Biomedical Co. Ltd., Suita, Japan). X-ray images were obtained using a μFX-1000 (Fuji Photo File Co. Ltd.). (c) Pedigrees of the TOY-KO mouse mated with C57BL/6J (shown as B6) and 129Sv mice are shown in the lower panel. Blue indicates a mouse with hydrocephalus, and green indicates a mouse carrying the causative mutation without the hydrocephalus phenotype.

To evaluate the contribution of 8-oxoG to *de novo* germline mutation, we established the TOY-KO mouse in the C57BL/6J background (>N16). TOY-KO mice are viable and fertile, although increased amounts of 8-oxoG accumulated in various tissues, including the gonads (Fig. 1a). Moreover, TOY-KO mice had a shorter lifespan (Fig. 1b) and developed various types of tumors (Fig. 1c). We maintained the TOY-KO mouse line originating from one pair (G1) to the 8th generation (G8) by intragenerational mating (Supplementary Fig. S2 online). More than 35% of TOY-KO mice carried macroscopically distinguishable tumors (Supplementary Fig. S2 online). As the generations increased, it became difficult to obtain mice for breeding because of the decreased number of weaned mice (Fig. 1d). Several phenotypic variations were found among the progeny, such as hydrocephalus, belly white spot and anophthalmia (Supplementary Fig. S2 online). In cases of hydrocephalus and white spot, the traits were transmitted to the next generation in an autosomal dominant fashion with incomplete penetrance (Fig. 2, Supplementary Fig. S2 online). These features indicate that heritable mutations could arise in the TOY-KO mice.

**Figure 3:**
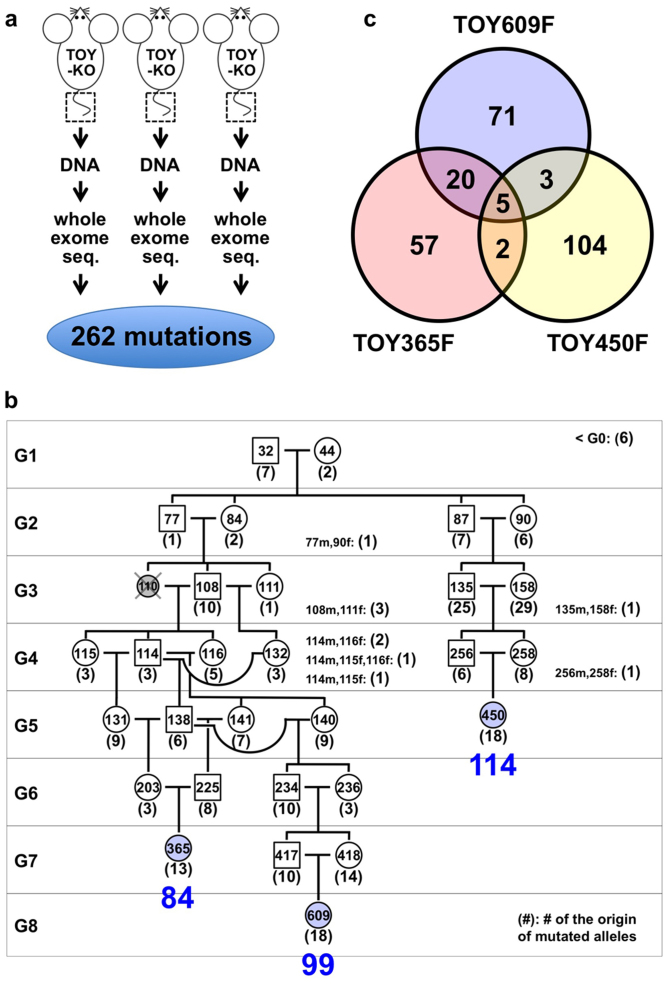
Identification of *de novo* germline mutations in TOY-KO mice. (a) Scheme for screening of germline mutations. (b) Pedigree of TOY-KO mice used for germline mutation analysis. TOY365F, TOY609F and TOY450F were used to identify *de novo* germline mutations. Blue numbers, 84, 98, and 114, indicate the number of mutations detected in TOY365F, TOY609F and TOY450F, respectively. Numbers in parentheses indicate the number of original mutations in each generation, which were found in tail DNA for the first time in the pedigree. The DNA of TOY110F was unavailable; therefore, the mouse was excluded from the analysis. (c) The numbers of base substitution mutations found in TOY365F, TOY609F and TOY450F.

To detect mutations that occur in the germ cell lineage and are transmitted across generations of TOY-KO mice, we performed whole exome sequencing analysis (Fig. 3a). We searched for different sequences between the C57BL/6J mouse reference genome (MGSCv37) and TOY-KO mice that belonged to the most advanced generation of each branch of the pedigree (TOY365F, TOY609F and TOY450F, shown in Fig. 3b). No sequencing reads corresponding to parts of the wild-type reference sequences of targeted *Mutyh, Mth1*, and *Ogg1* loci were obtained in chromosomes 4, 5, and 6, respectively (Supplementary Fig. S3 online), which confirmed that the TOY-KO mouse was indeed deficient for the three genes, and validated our exome analysis. By analyzing the exome covering 40.9 Mb of mouse transcribed sequences, which included 19,427 genes from 17 chromosomes, excluding chromosomes 4, 5, and 6 from the analysis to avoid ambiguity, we identified 262 base substitution mutations (Fig. 3c, Supplementary Table S1 online, Supplementary Data S1 online). No insertion/deletion mutations were detected in this analysis.

### Identification of mutation origin mice

**Figure 4:**
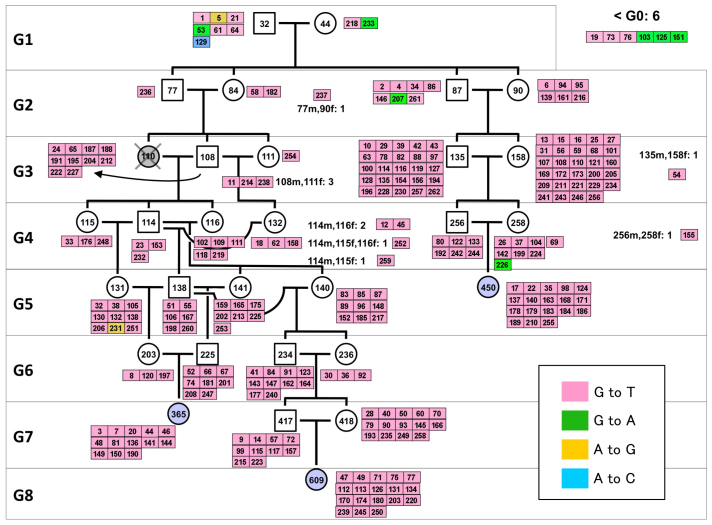
Heritable mutations mapped in the pedigree of TOY-KO mice. The number in each box indicates the mutation ID number shown in Supplementary Data S1 online, and the color indicates the mutation category.

**Figure 5:**
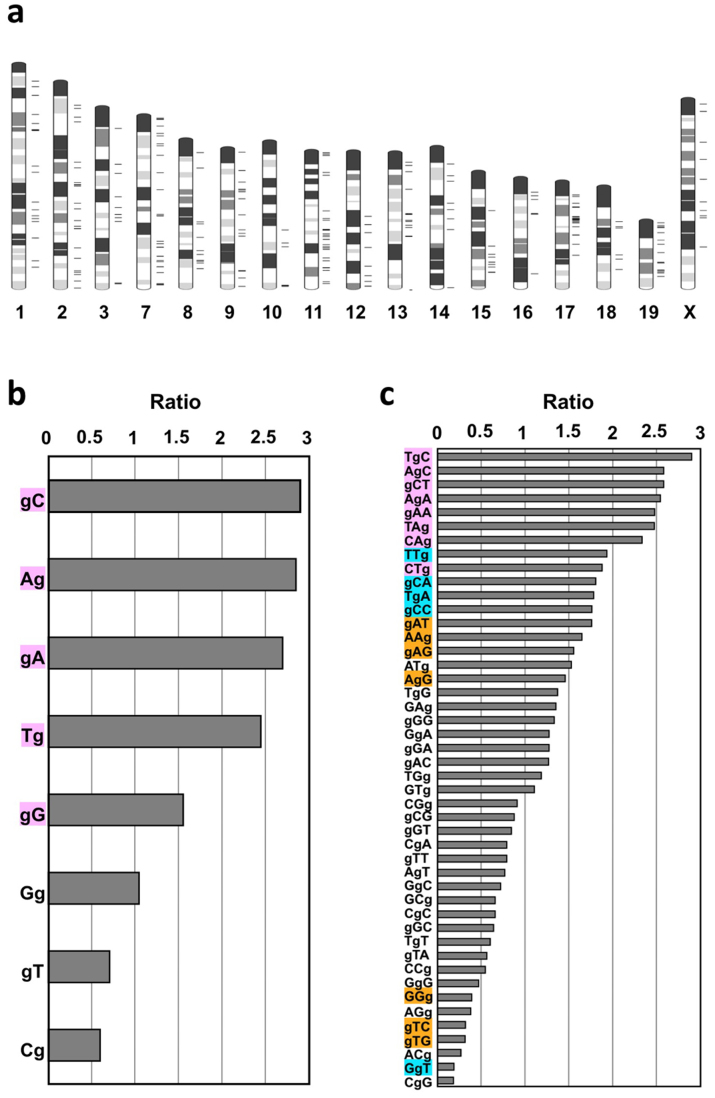
Genome-wide distribution of mutations and site preferences of G to T mutations in di- and trinucleotide sequences. (a) Mutations detected in G2–G8 were mapped on a mouse G-band ideogram using Ideographica (http://www.ncrna.org/idiographica/). Each black transverse line on the right side of the chromosome represents a mutation site. (b) Site preferences of G to T mutations in di-nucleotide sequences. The plots represent the relative ratio of the actual value of detected mutations (G to T mutations in G2–G8) in each di-nucleotide to its occurrence level in the analyzed exome sequences. ‘g' indicates the position of a mutated guanine. (c) Site preferences of G to T mutations in tri-nucleotides. For each nucleotide sequence, a chi square test (detected vs. expected) was performed, and the colored sequences indicate a significant difference: *P* < 0.001 (pink), *P* < 0.01 (blue), and *P* < 0.05 (orange).

The 262 mutations detected in TOY365F, TOY609F and TOY450F had occurred in one of the mice in the 8-generations of the pedigree (Fig. 3b); therefore, we determined the mutation origin mouse that initially possessed the mutated allele in its tail DNA. We traced each mutation on the pedigree by determining the sequences of all mutated alleles in 35 TOY-KO mice shown in the pedigree (Fig. 3b), using MassArray or Sanger's sequencing, and identified the origin of each *de novo* mutation. The results of the sequencing are summarized in Supplementary Data S1 online with annotations. Among them, we considered that 247 mutations found in G2–G8 mice had spontaneously occurred in the germ cell lineage of TOY-KO mice, because these mutated alleles were derived from gametes of their parent mice (G1–G7) or were generated during early development of the mice (G2–G8). The spectrum of germline mutation observed in TOY-KO mice indicated a distinct feature: 99% (244/247) of the mutations were G to T transversions (Table 1). G to T mutations had specifically increased in TOY-KO mice lacking the ability to avoid 8-oxoG-induced mutations; therefore, we concluded that 8-oxoG is a causative molecule for spontaneous G to T mutation in the mouse germ cell lineage. These mutations arose in all progeny of each generation and in all chromosomes that we analyzed (Figs. 4 and 5a). The mutations ranged from synonymous substitutions to harmful mutations, such as a gain of a stop codon in the *Ttn* gene responsible for human hypertrophic cardiomyopathy^18^ (Supplementary Data S1 online).

By analyzing the position of the mutated G in di- and tri-nucleotide sequences, we found that G to T mutations occurred more often at GpC sites than at CpG sites, and tended to occur at tri-nucleotides, which are typical sequences found in triplet repeat expansion disorders (Fig. 5b, c), such as CAG (Huntington's disease), CTG (Myotonic dystrophy) and GAA (Friedreich ataxia)^19^. It is probable that uneven distribution of mutable 8-oxoG is reflected by the tendency for DNA oxidation, or by the site preference of DNA polymerases in incorporating 8-oxodGTP. We also detected two G to A and one A to G transition mutations that were classified as synonymous coding or intronic mutations (Table 1, Supplementary Data S1 online).

### *De novo* germline mutation rate of TOY-KO mouse

The detected mutations accumulated in TOY365F, TOY450F and TOY609F contained parts of the mutations that had occurred in the germ cells of the ancestral mice, because only half of the chromosomes derived from the father and mother had transmitted to the offspring via gametogenesis and fertilization in each generation. The numbers of newly arisen mutations detected only in TOY365F, TOY450F and TOY609F were 13, 18 and 18, respectively (Fig. 3b). Therefore, the *de novo* germline mutation rate was calculated to be 2.0 × 10^−7^/base/generation (13 + 18 + 18/3/40.9 Mb × 2/generation). This mutation rate is 18-fold higher than the basal level, 1.1 × 10^−8^ mutations/base/generation, calculated from the specific locus test in mice^20^. For human trio analysis^1^, the germline mutation rate was calculated to be 1.2 × 10^−8^ mutation/base/generation, and the G to T transversion mutation was observed in about 9% of all mutations. These results indicated that an approximately 200-fold increase in G to T transversion mutations occurred in the TOY-KO mice. No G to A transition mutations occurred in TOY365F, TOY450F, and TOY609F (totaling 245.4 Mb); therefore, the background mutation level of the TOY-KO mouse was estimated to be less than 4.1 × 10^−9^ G to A transition mutation/base/generation. This background mutation level is not high compared with that in humans (4.9 × 10^−9^ G to A transition mutation/base/generation)^1^.

### Fates of *de novo* germline mutations

**Figure 6:**
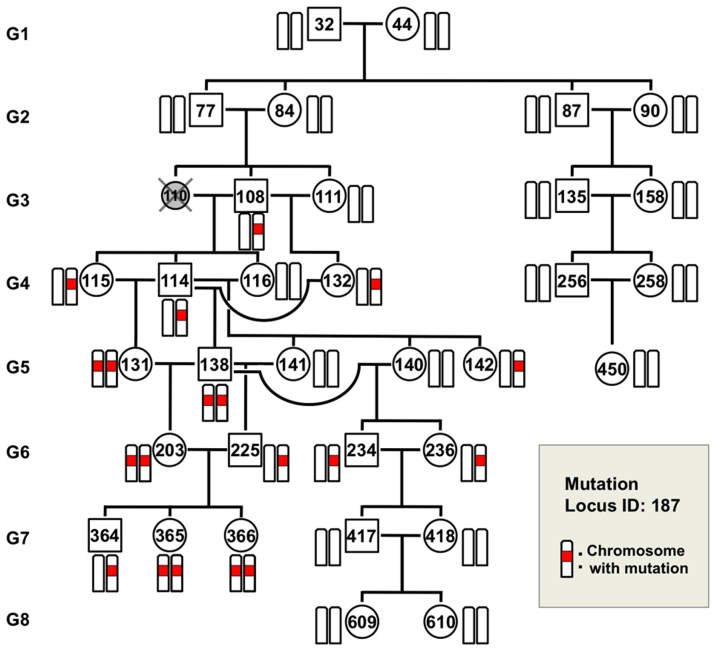
Fate of a germline mutation. Mutation #187 (Ch. 15) was chosen to show the fate of a mutation generated in TOY-KO mice through the generations. This mutation initially appeared in TOY108M (G3) as a heterozygous allele. It was transmitted to progeny TOY-114M, TOY-115F, and TOY-132F. At G5, mutation #187 became homozygous in TOY138M and TOY131F, and thus were fixed in the progeny. Conversely, in another branch, the mutation was not transmitted from TOY-234M and TOY-236F (G6) to their offspring and eventually disappeared. The mutated locus is indicated in red.

By following up the mutated alleles in the pedigree, we observed the fates of the *de novo* mutations, in which some were fixed and others were eliminated in later generations. As shown in Fig. 6, for example, mutation #187 initially appeared in TOY108M (G3) as a heterozygous allele, indicating that the mutation probably occurred in the germ cell lineage of the parents, either TOY77M or TOY84F, and was transmitted to the progeny. At G5, it became homozygous in TOY138M and TOY131F, and thus fixed in the progeny. Conversely, in another branch, the mutant allele was not transmitted to the offspring and eventually disappeared. These behaviors of the mutated allele represent the appearance, transmission, fixation and disappearance of a spontaneous mutation, which are the typical fates of a novel mutation in the evolutionary process.

## Discussion

Little research has been performed to identify the causative molecule of spontaneous germline mutations because it is a rare event. We considered that the causative molecule must possess certain features that make DNA more prone to mutation, be generated endogenously and spontaneously and remain in the germ cell lineage. In 2006, we reported that endogenous 8-oxoG is distributed in the genome of human lymphocytes in the steady state^5^. We hypothesized that 8-oxoG also exists in the genome of germ lineage cells, and is responsible for spontaneous *de novo* germline mutations, because 8-oxoG is endogenously generated by ROS derived from cellular respiration, and is known to cause transversion mutations. By disruption of the 8-oxoG exclusion system in mice, we detected increased spontaneous accumulation of germline mutations during the generations. These mutations were distributed throughout the chromosomes and inheritable to offspring across the generations, leading to an expansion of genetic diversity as well as disease-associated mutations.

The effects of 8-oxoG on spontaneous germline mutations were apparent in the TOY-KO mice. However, the production of 8-oxoG is dependent on the oxidation of guanine nucleotides, which occurs even in the wild-type cells independently of MTH1, OGG1 and MUTYH activities. It is likely that 8-oxoG universally causes *de novo* G-T transversion mutations, including germline mutations, although most of these mutations are efficiently prevented by the MTH1, OGG1 and MUTYH enzyme system.

**Figure 7:**
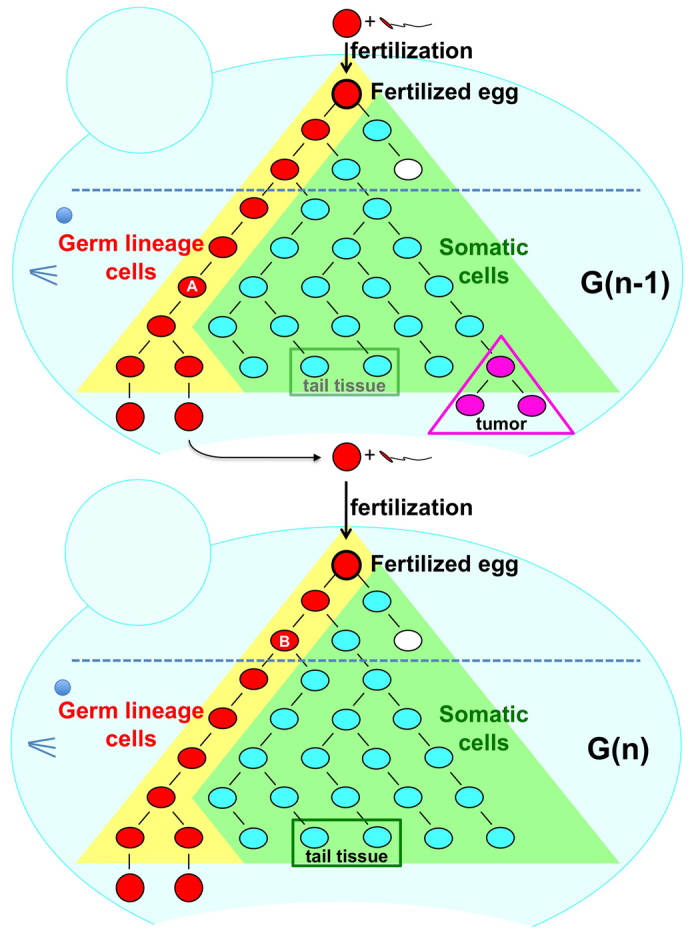
Germ line mutations occur at different stages of the germ cell lineage. Mutations detected in the tail DNA of the first mutant mouse had occurred either in the germ lineage cells of the previous generation or during the very early developmental stage of the mutant mouse. Mutations start to accumulate from the first replication of fertilized egg DNA; however, each mutation is diluted out in the tissue DNA. Therefore, we used the tail DNA sequence as a reference sequence of fertilized egg DNA. In contrast to tail tissue, differentiated gametes can transmit their sequence information monoclonally to offspring. If the original mutated allele was mapped in multiple mice of the same generation, such as mutation #54 (in Fig. 4, Supplementary Data S1 online), the mutation probably occurred in the germ lineage cells of the parents (indicated as A). For mutations in the X chromosome (such as mutation #261), which began in the male with a heterozygous status (see Supplementary Fig. S4 online), the mutation probably occurred in a cell during the early stage of embryonic development (shown as B), resulting in mosaicism of tail tissue. These results indicate that germline mutations occur at different developmental stages of the germ cell lineage.

When did the germline mutations occur? It is difficult to determine the timing of the occurrence of a mutation in the germ cell lineage; however, some examples were obtained that allowed us to speculate on the timing of mutations in our experiment. *De novo* mutations occur either in the germ cell lineage of the previous generation or during the very early developmental stage of the mutant mouse (Fig. 7). In eleven cases among 247 mutations, the mutations had likely occurred in the germ cell lineage of the parents, because the original mutated allele was detected in multiple mice of the same generation (Fig. 3b). For three mutations on the X chromosome (Mutation ID #257, #261 and #262), which began in males with a heterozygous status (Supplementary Fig. S4 online), the mutation probably occurred in a cell at an early stage of embryonic development, resulting in mosaicism of tail tissue. These results showed that the germline mutations occurred at different developmental stages of the germ cell lineage. It is noteworthy that most germline mutations occurred during mitoses, because the germ cell lineage from fertilized egg to differentiated sperm or egg requires a large number of mitoses and only one meiosis. In the other cases (233/247) shown in Fig. 3b (G2–G8), the original mutated allele was found in a single mouse of each generation, and we could not identify when the mutation occurred.

By analogy to the *Escherichia coli* system, we considered that 8-oxoG-induced G to T mutation is suppressed by OGG1, MUTYH, and MTH1, whereas the A to C mutation is prevented by MTH1 in mammalian cells (Supplementary Fig. S5 online). However, in contrast to the *E. coli* *mutT, mutM, mutY* triple mutant, in which both G to T and A to C mutations increased^21^, no A to C germline mutations were detected in the TOY-KO mouse. Thus, it is likely that different mechanisms, such as mismatch repair^22^ or proof reading by DNA polymerase, may function to avoid A to C mutations caused by 8-oxodGTP in the TOY-KO mouse, even in the absence of MTH1. It has been reported that 2-hydroxy-deoxyadenosine (2-OHdA), an oxidized form of deoxyadenosine, is recognized as a substrate by the MUTYH protein and possesses premutagenic features^23^,^24^. 2-OHdATP, a triphosphate form of 2-OHdA, is a substrate of the MTH1 protein^25^. The MutY and MutT proteins of *E. coli* cannot recognize 2-OHdA, in contrast to the mammalian enzymes^24^,^26^. At the present, we cannot evaluate the contribution of 2-OHdA to the increase of germline mutation observed in TOY-KO mice, because we have not yet confirmed the accumulation of 2-OHdA in the DNA. Thus, the significance of 2-OHdA for germline mutations remains to be elucidated.

The TOY-KO mouse strain spontaneously accumulates mutations in the homozygous status. For genome-wide screening of mutants, this mouse has unique features and has the potential to take a complementary role to ENU mutagenesis^27^,^28^. The mutation is specific for G to T transversions, and occurs spontaneously and continuously in both male and female germ lineage cells of TOY-KO mice. The mutation rate of TOY-KO mice (0.2 mutation/Mb/generation, on average, in male and female) is lower than ENU-treated male gametes (1 mutation/0.42–1.82 Mb for male mouse^27^, 1 mutation/3.7 Mb in male rat^28^); however, the number of mutations carried by each TOY-KO mouse increased as the generations increased. Similar to ENU mutagenesis, phenotype-driven screening is available. Currently, the TOY-KO mouse is only available in the C57BL/6J genetic background; however, it would be a good system for large genome-wide screening of dominant mutations. Using such mutator mice with a well-controlled genetic background would permit the evaluation of the contribution of aging and the difference between spermatogenesis and oogenesis on the accumulation of germline mutations. This system also enables us to assess the genotoxic effects of chemical and environmental factors on mammalian germ lineage cells.

Although *de novo* germline mutations cause sporadic genetic diseases in humans, their occurrence is an important step for the evolution of species, as well as selection for survival. 8-oxoG, one of the causative molecules of these mutations, is endogenously produced by ROS generated from biological processes, such as oxygen respiration and inflammation, and is widely present in the DNA of various organisms. It is likely that the oxidative environment expands the genetic diversity of species by increasing the mutation rate of the germ lineage cells to accelerate the evolutionary process. MTH1, OGG1 and MUTYH, which are well conserved among species, may have contributed coordinately to control the germline mutation rate to an appropriate level for each species during evolution by controlling the amount of 8-oxoG in the genome (Supplementary Fig. S1 online).

## Methods

### Animals

*Mth1*^+*/−*^, *Ogg1^+/−^*, and *Mutyh^+/−^* mice were established^13^,^14^,^16^ and backcrossed to C57BL/6J:Jcl (Clear Japan, Tokyo, Japan) for more than 16 generations. By crossing the C57BL/6J-background *Ogg1^+/−^, Mth1*^+*/−*^, and *Mutyh^+/−^* mice, we obtained *Mth1^+/−^/Ogg1^+/−^* mice and *Ogg1^+/−^/Mutyh^+/−^* mice. *Mth1^+/−^/Ogg1^+/−^* mice were then mated with *Ogg1^+/−^/Mutyh^+/−^* mice to obtain *Mth1^+/−^/Ogg1^−/−^/Mutyh^+/−^* mice. Finally, by crossing the *Mth1^+/−^/Ogg1^−/−^/Mutyh^+/−^* mice, we obtained a pair of *Mth1^−/−^/Ogg1^−/−^/Mutyh^−/−^* mice (TOY32M and TOY44F). All animals were maintained in a temperature-controlled (22 ± 2°C, 55 ± 5% humidity), specific pathogen-free room with a 12-h light-dark cycle. The care and use of all animals were performed in accordance with prescribed national guidelines, and the Animal Care and Use Committee of Kyushu University granted ethical approval for the study.

### Quantification of 8-oxo-dG by LC-MS/MS

To detect the level of nuclear 8-oxodG, C57BL/6J:Jcl and TOY-KO mice (12–14 weeks old) were euthanized by cervical dislocation, and tissues were immediately removed and frozen in liquid nitrogen. DNA was extracted using a DNA Extractor TIS Kit (# 296-67701, Wako Pure Chemical Industries, Osaka, Japan), according to the manufacturer's instructions, with a slight modification: 10 mM 2, 2, 6, 6-tetramethylpiperidine-N-oxyl (Wako Pure Chemical Industries) was added to all reagents at all stages of manipulation^29^. Extracted DNA was hydrolyzed with 0.17 mg/ml nuclease P1 (Yamasa, Chiba, Japan) and 1.7 μM acid phosphatase (P-1435, Sigma-Aldrich Japan Inc., Tokyo, Japan) in 17 mM sodium acetate buffer (pH 4.5) at 37°C for 30 min, followed by filtration at 12,000 × *g* for 3 min (Ultrafree-MC probind 0.45 μm, Millipore, Billerica, MA). The digested samples (100 μl) were subjected to liquid chromatography-tandem mass spectrometry (LC-MS/MS) analysis using a Shimadzu VP-10 HPLC system connected to an API3000 MS/MS system (PE-SCIEX, SpectraLab Scientific Inc, Ontario, Canada).

### Statistical analyses

Statistical analyses were conducted using JMP 9.02 (SAS Institute Japan, Tokyo, Japan).

### Detection of germ line mutations by whole exome sequencing

Exome sequencing libraries for three TOY-KO mice (TOY365F, TOY450F and TOY609F) and five DBF1 (DBA/2J:Jcl × C57BL/6J:Jcl F1) mice as controls were prepared using a SureSelect^XT^ Mouse All Exon Kit (Agilent Technologies Japan, Tokyo, Japan), according to the manufacturer's instructions. Briefly, 3 μg of genomic tail DNA was sonicated into 150–180 bp fragments using a Covaris S2 System (Covaris, Woburn, MA, USA). The adaptors were ligated to the sonicated DNA after blunting and ~200 bp fragments were extracted using a 2% E-Gel (Life Technologies Japan, Tokyo, Japan). The extracted fragments were amplified with 2.5 mM SureSelect Pre-Capture primers and Platinum PCR Amplification Mix (Life Technologies), under the following conditions: 72°C for 20 min and 95°C for 5 min; 12 cycles of 95°C for 15 sec, 54°C for 45 sec and 70°C for 1 min; and a final extension at 70°C for 5 min. The PCR products were purified with a PureLink column (Life Technologies Japan). Purified PCR products (500 ng) were hybridized for 36 h at 65°C with SureSelect baits, according to the manufacturer's protocol. The captured libraries were amplified with the SureSelect Barcoding primer (BC1-8) for SOLiD with Herculase II Fusion DNA Polymerase (Agilent Technologies Japan), under the following conditions: 95°C for 5 min; 8 cycles of 95°C for 15 sec, 54°C for 45 sec and 70°C for 1 min; final extension at 70°C for 5 min. The captured barcoding libraries were quantified with an Agilent QPCR NHS Library Quantification Kit (Agilent Technologies Japan) and pooled. The four pooled libraries (1 pM) were amplified and purified with an EZ bead system (Life Technologies Japan). Purified P2-enriched beads were sequenced on one full slide of a SOLiD4 system (Life Technologies Japan). About 130 million paired-end sequencing reads (50 bp and 35 bp) were obtained from each library. Bioscope1.3.1 (Life Technologies Japan) was used to map the SOLiD paired-end reads to the mm9 reference mouse genome sequence (MGSCv37) using default parameters for Targeted resequencing methods. BEDtools v2.16.2 were used to calculate the coverage depth statistics and target enrichment efficiency. Avadis-NGS v1.3 (Strand Scientific Intelligence Inc., Karnataka, India) was used to carry out single nucleotide variant (SNV) calling with eight BAM format files (three TOY-KO lines and five control samples). The cutoff parameters of the SNV call were as follows: filtered sequencing quality ≤20, filtered PCR duplications, consensus base quality ≤50, total coverage <10, variants read depth <3, and the Decibel Score by Avadis-NGS v1.3 <50. The Decibel Score, read depth of the SNV allele and SNV allele frequency were used to sort these candidates. The iterative genomic viewer was used to check the candidates sequentially to eliminate apparent false positives. Finally, MassARRAY was used to select 286 mutation candidates for validation experiments (Supplementary Table S1 online).

### Confirmation of mutations by sequencing

A MassARRAY3 Analyzer (Sequenom Inc, San Diego, CA) with iPLEX Gold Genotyping Reagent (Sequenom Inc) was used to validate the 286 candidates, according to the manufacturer's instructions. Briefly, MassARRAY Typer4 Assay Designer (Sequenom Inc) designed the 286 PCR primer pairs and 286 iPLEX primers as single-base extension primers for each candidate. We used 37 genomic DNA samples, including 35 samples from the TOY-KO pedigree and two control samples, as well as C57BL/6J and the original ES cell DNA to determine the origin of the *de novo* mutations in the TOY-KO pedigree. Ten nanograms of genomic DNA were used in each multiplex PCR for the MassARRAY. After dephosphorylation, single-base extension with the iPLEX primer and desalting were performed. The reaction products were spotted onto a 384-format SpectroCHIP with a MassARRAY Nanodispenser (Sequenom Inc) and then subjected to a MassARRAY 3 analyzer (Sequenom Inc). MassARRAY Typer 4.0 software (Sequenom Inc) was used to analyze the mass spectrum data.

### Determination of the site preference of G to T mutation in di- and trinucleotide sequences

To analyze site preference of G to T mutation caused by *Mth1/Ogg1/Mutyh* deficiency, the 239 data of G to T mutation detected in G2–G8 were subjected (C to A mutations were converted to G to T mutation). The reference exon sequences and the 101 nucleotides those containing each mutation site (shown in Supplementary Data S1 online) were used to determine the site preference of mutation. The ratio shown in Fig. 5b, c were calculated as follows (data were summarized in Supplementary Table S2 online).

- The number of each di- or tri-nucleotides sequences in the reference exon sequence were counted by 1 nucleotide sliding.
- The number of each di- or tri-nucleotides sequences that include mutated guanine site were counted.
- The frequency of each di- or tri-nucleotides sequences was calculated as follows: (A) /number of total nucleotide in reference exon sequence.
- Total number of di- or tri-nucleotides sequences that include mutated guanine site were 478 and 717, respectively.
- The expected value for a random mutation for each di- or tri-nucleotides sequences were calculated as (C) × (D).
- The ratio (observed mutation for the expected value for a random mutation) was calculated as (B)/(E).

## Author Contributions

M.O. and K.S. designed the study, analysed data, and wrote the paper. R.F. and Y.G. designed and performed exome and sequencing analyses. Y.I. and T.I. performed bioinformatic analysis. T.T. gave conceptual advice. M.F. quantitated 8-oxodG. M.H. analysed hydrocephalus mice. Y.N. was involved in the study design and preparation of the paper. All authors discussed the results and commented on the manuscript.

## Supplementary Material

Supplementary Information Supplementary information

Supplementary Information Dataset 1

## Figures and Tables

**Table 1:** Spectrum of heritable mutations in TOY-KO mice

	All	G2–G8
G:C to A:T	7	2
A:T to G:C	2	1
G:C to T:A	252	244
A:T to C:G	1	0
G:C to C:G	0	0
A:T to T:A	0	0
total	262	247

Mutations detected in the 40.9 Mb exome sequences of TOY365F, TOY450F, and TOY609F (Fig. 3a) were classified into mutation types. The mutations observed in G2–G8 mice (Fig. 3b) were considered as mutations that occurred in the TOY-KO germ cell lineage.
