# Supplementary material for: 8-oxoguanine causes spontaneous de novo germline mutations in mice
Source: Sci Rep. 2014 Apr 15;4:4689. doi: 10.1038/srep04689 (PMC3986730; doi:10.1038/srep04689)
Supplement: Supplementary Information [file srep04689-s1.pdf]

Supplementary information

Title: 8-oxoguanine causes spontaneous *de novo* germline mutations in mice

Mizuki Ohno[1], Kunihiko Sakumi[2,3,*], Ryutaro Fukumura[4], Masato Furuichi[5], Yuki Iwasaki[6,7], Masaaki Hokama[2], Toshimichi Ikemura[6], Teruhisa Tsuzuki[1], Yoichi Gondo[4], Yusaku Nakabeppu[2,3]

8-oxoG induced G to T transversion mutation in germ lineage cells

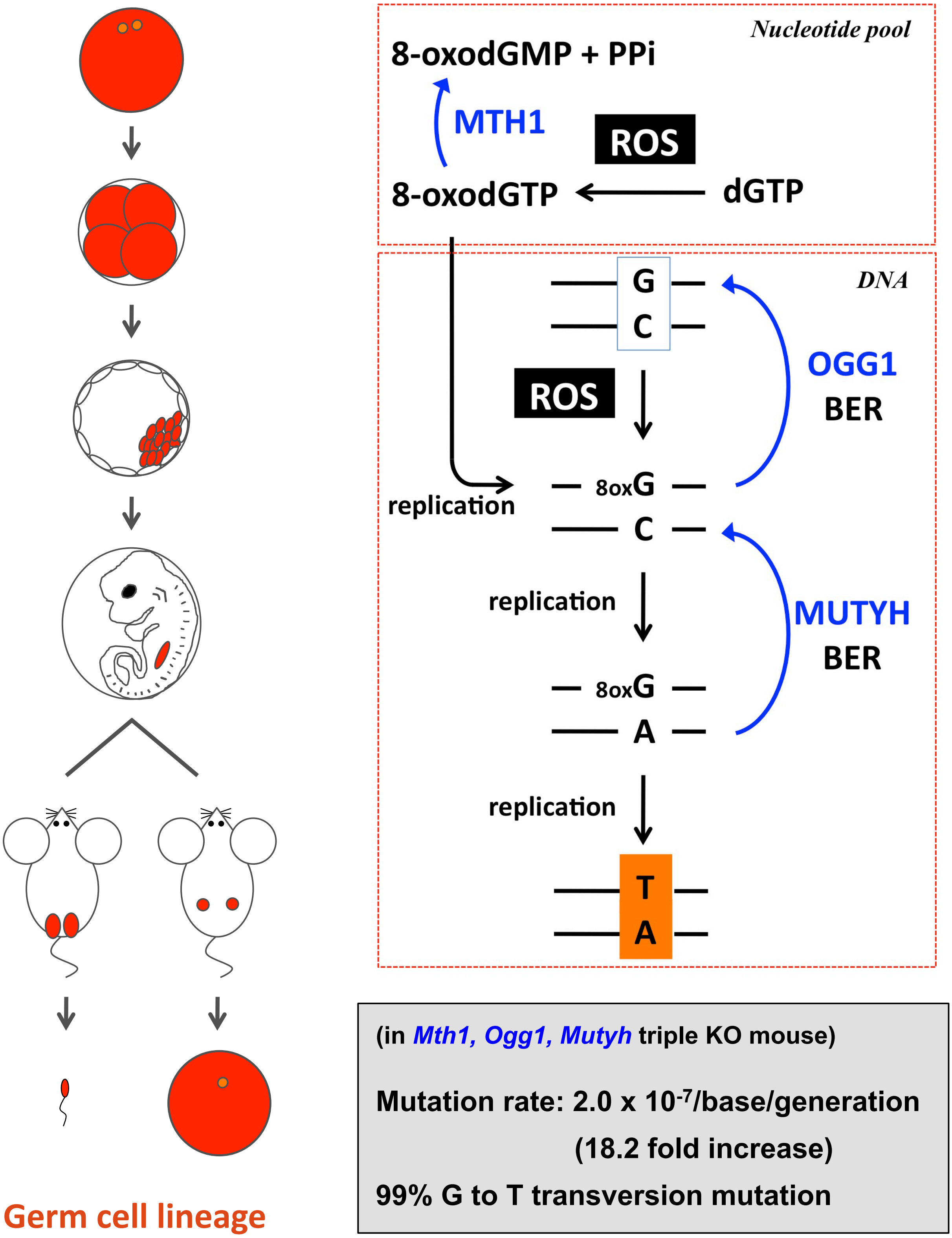

Supplementary Fig. S1

a

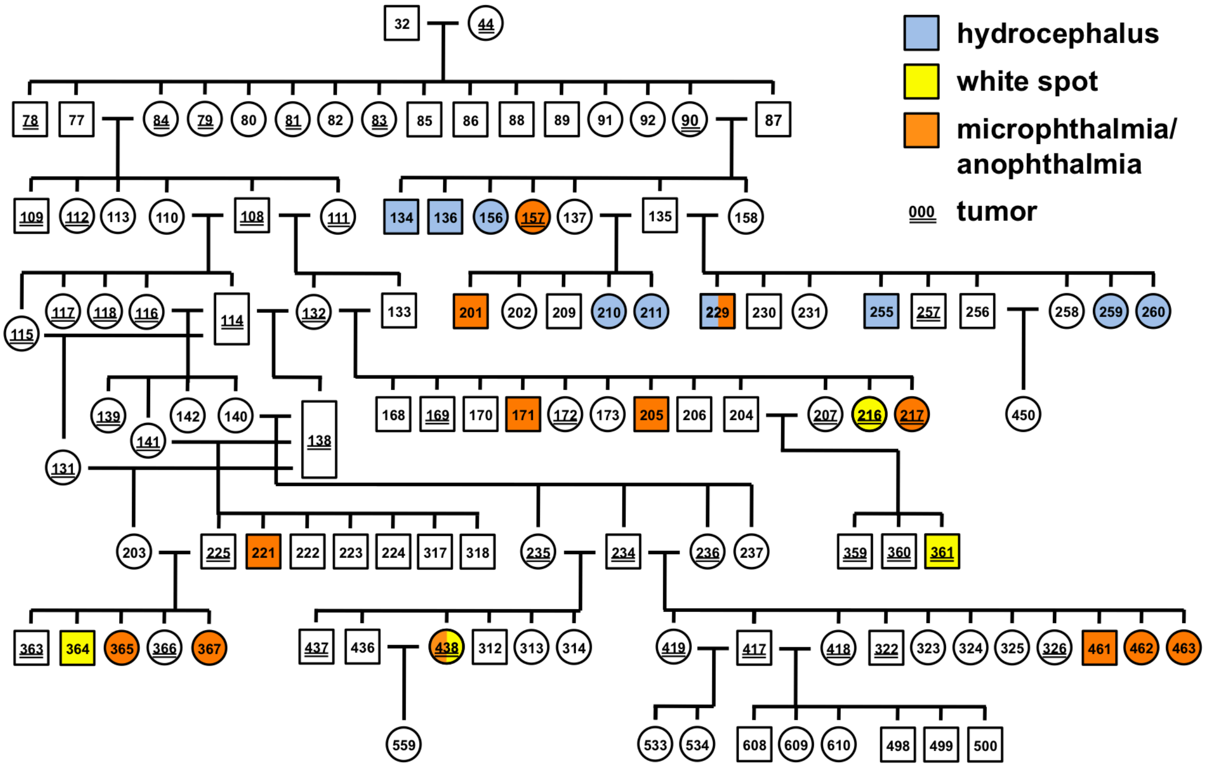

b

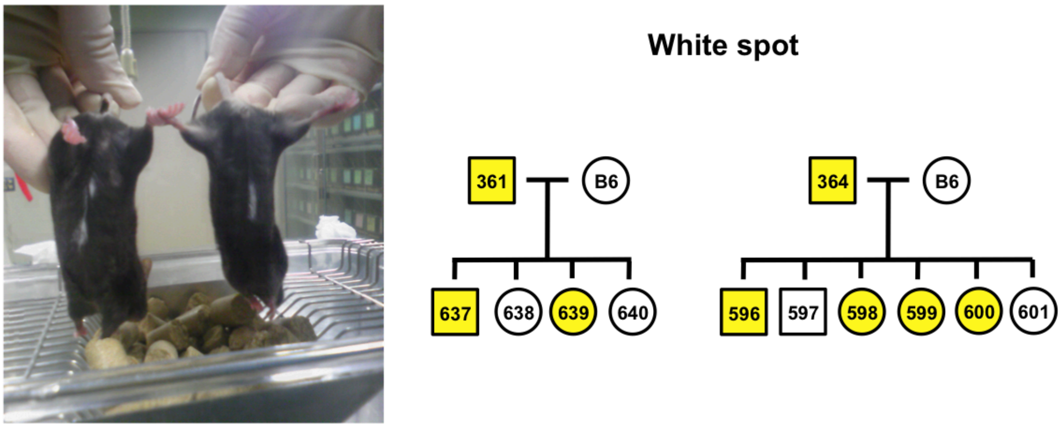

c

Anophthalmia (left) and microphthalmia (right)

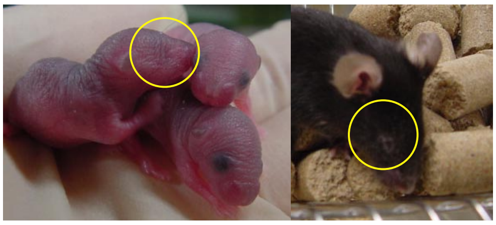

Supplementary Fig. S2

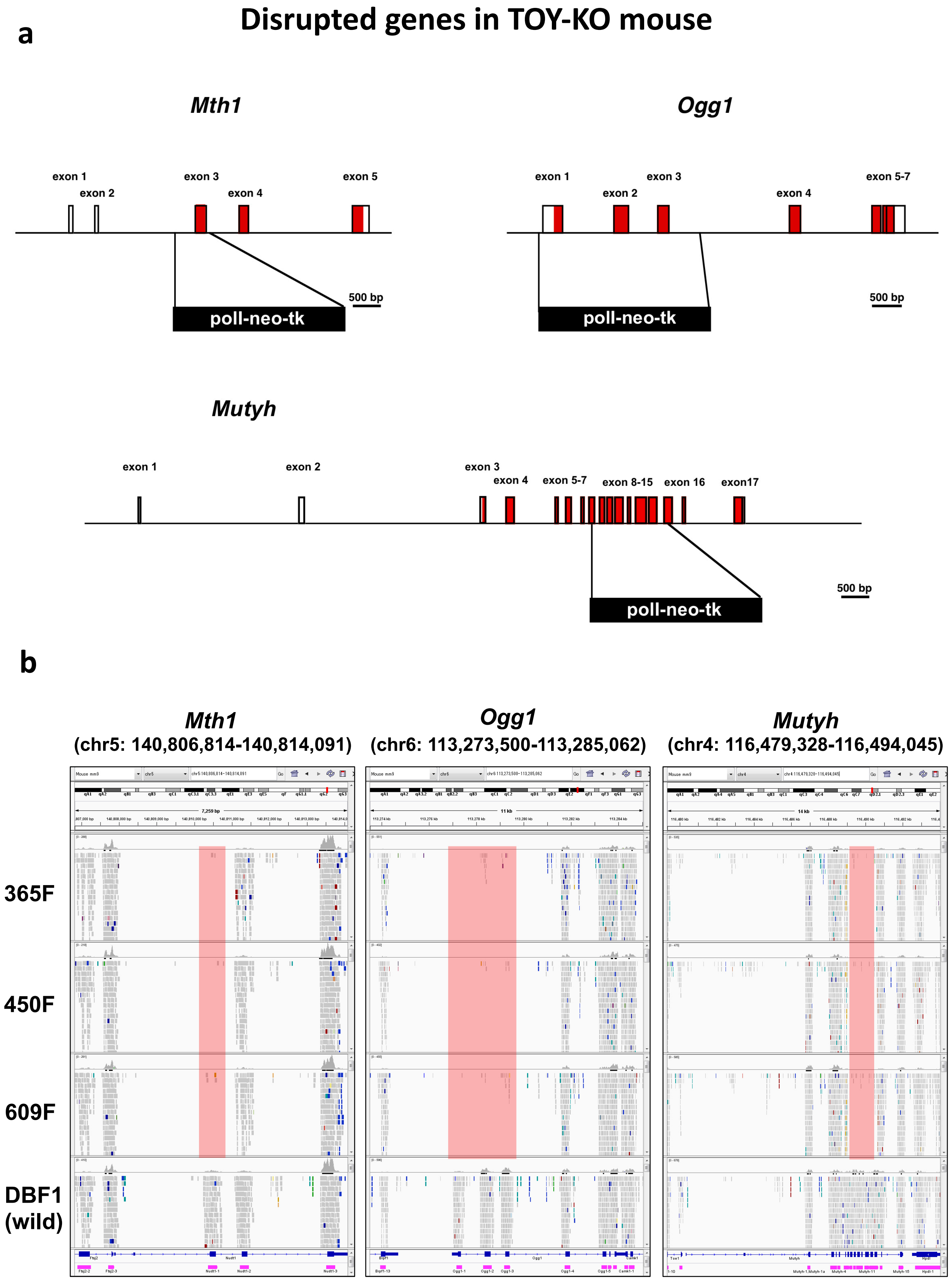

Supplementary Fig. S3

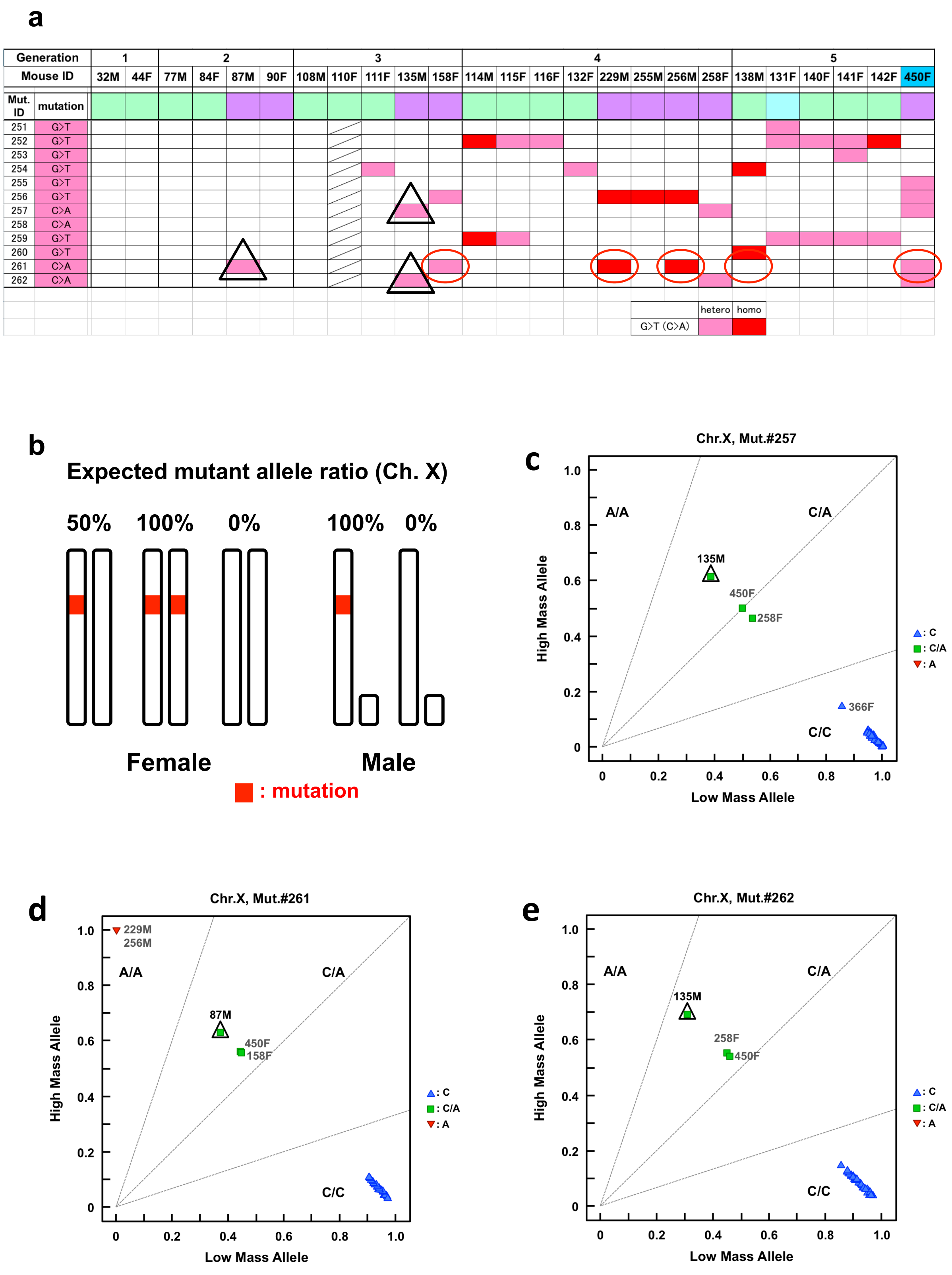

Supplementary Fig. S4

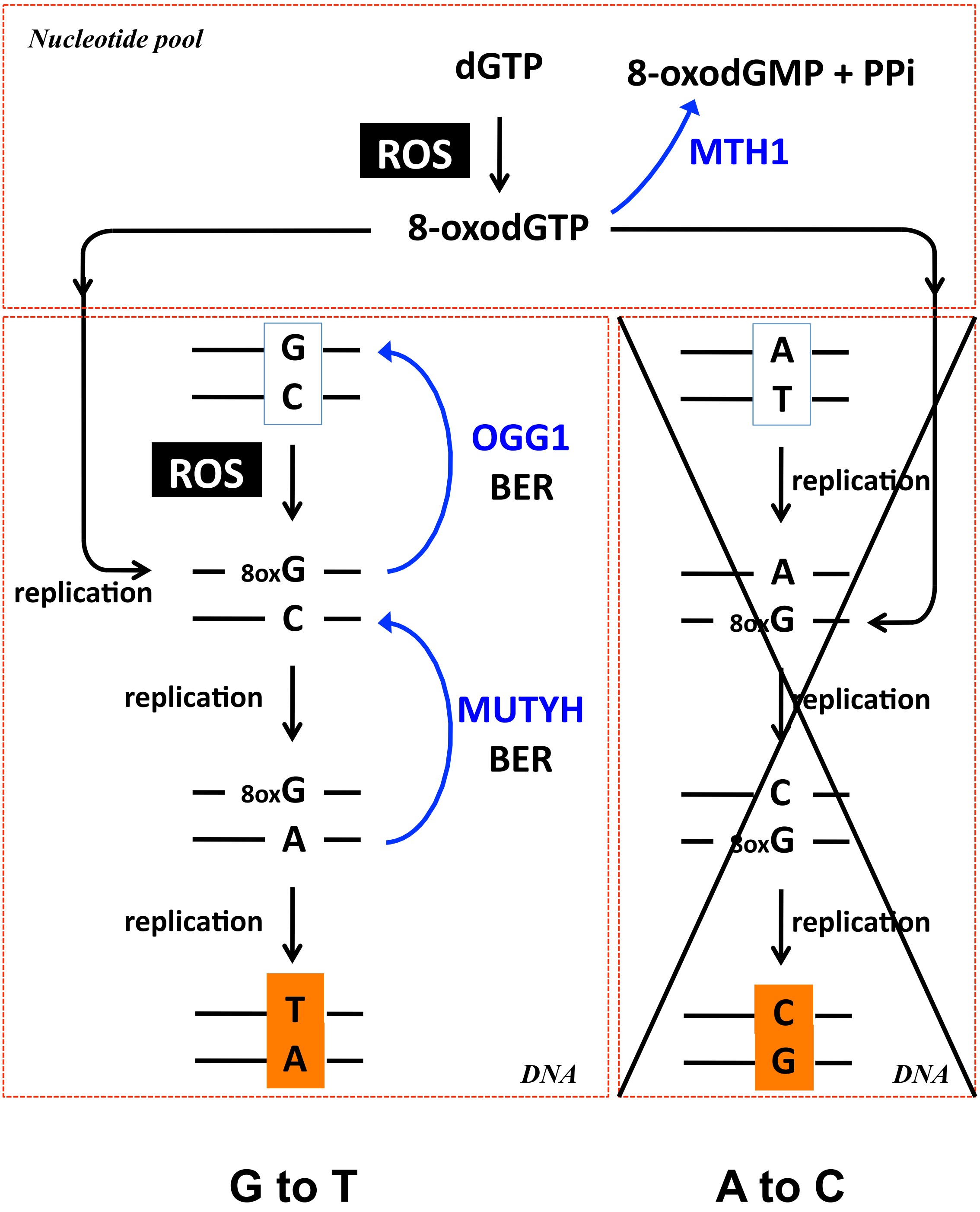

8oxG: 8-oxoG

ROS: reactive oxygen species

BER: base excision repair

Supplementary Fig. S5

Supplementary Table S1 Extraction and validation of mutation candidates from TOY-KO mice.

	TOY 365F	TOY 450F	TOY 609F	TOY 365F & 450F	TOY 365F & 609F	TOY 450F & 609F	TOY 365F & 450F & 609F	total
**Mutation candidates by Avadis-NGS**	**1,278**	**1,242**	**1,504**	**51**	**28**	**20**	**7**	**4,130**
**Extracted candidates**	**66**	**108**	**73**	**7**	**23**	**3**	**6**	**286**
**Validated mutations**	**57**	**104**	**71**	**2**	**20**	**3**	**5**	**262**

Upper row indicates numbers of mutation candidates initially screened by calculation of each BAM file with Avadis-NGS v1.3. Middle row shows numbers of further selected candidates by higher stringent conditions. Lower row shows numbers of confirmed mutations validated with MassARRAY sequencing analysis.

Supplementary Table S2 Determination of the site preference ratio of G to T mutation in di- and trinucleotide sequences.

	(A):The number of each di- or tri-nucleotides sequences in the reference exon sequence	(B): The number of each di- or tri-nucleotides sequences that include mutated guanine site	number of total nucleotide in reference exon sequence	(C): The frequency of each di- or tri-nucleotides sequences : (A) / number of total nucleotide in reference exon sequence	(D): Total number of di- or tri-nucleotides sequences that include mutated guanine site were 478 and 717, respectively.	(E): The expected value for a random mutation for each di- or tri-nucleotides sequences	(F): The ratio (observed mutation for the expected value for a random mutation) was calculated as follows: (B)/(E)
Ag	6872920	106	88412828	0.07774	478	37.15813	2.85267
Cg	2175522	7	88412828	0.02461	478	11.76186	0.59514
Gg	6194524	35	88412828	0.07006	478	33.49042	1.04508
Tg	6878679	91	88412828	0.07780	478	37.18927	2.44694
gA	5828534	85	88412828	0.06592	478	31.51171	2.69741
gC	5367424	84	88412828	0.06071	478	29.01874	2.89468
gG	6194524	52	88412828	0.07006	478	33.49042	1.55268
gT	4719951	18	88412828	0.05339	478	25.51820	0.70538
AAg	1795386	24	88412828	0.02031	717	14.56001	1.64835
ACg	461200	1	88412828	0.00522	717	3.74019	0.26737
AGg	1945305	6	88412828	0.02200	717	15.77581	0.38033
ATg	1454699	18	88412828	0.01645	717	11.79715	1.52579
CAg	2429511	46	88412828	0.02748	717	19.70256	2.33472
CCg	677091	3	88412828	0.00766	717	5.49099	0.54635
CGg	677091	5	88412828	0.00766	717	5.49099	0.91058
CTg	2429511	37	88412828	0.02748	717	19.70256	1.87793
GAg	1825637	20	88412828	0.02065	717	14.80534	1.35086
GCg	563085	3	88412828	0.00637	717	4.56644	0.65697
GGg	1572721	5	88412828	0.01779	717	12.75427	0.39203
GTg	1564626	14	88412828	0.01770	717	12.68862	1.10335
TAg	797186	16	88412828	0.00902	717	6.46493	2.47489
TGg	1978359	19	88412828	0.02238	717	16.04386	1.18425
TTg	1403774	22	88412828	0.01588	717	11.38416	1.93251
AgA	1940268	40	88412828	0.02195	717	15.73496	2.54211
AgC	1673047	35	88412828	0.01892	717	13.56788	2.57962
AgG	1945305	23	88412828	0.02200	717	15.77581	1.45793
AgT	1284533	8	88412828	0.01453	717	10.41716	0.76796
CgA	468238	3	88412828	0.00530	717	3.79726	0.79004
CgC	563085	3	88412828	0.00637	717	4.56644	0.65697
CgG	677091	1	88412828	0.00766	717	5.49099	0.18212
GgA	1741070	18	88412828	0.01969	717	14.11953	1.27483
GgC	1540454	9	88412828	0.01742	717	12.49259	0.72043
GgG	1572721	6	88412828	0.01779	717	12.75427	0.47043
GgT	1314059	2	88412828	0.01486	717	10.65660	0.18768
TgA	1659508	24	88412828	0.01877	717	13.45808	1.78331
TgC	1572329	37	88412828	0.01778	717	12.75109	2.90171
TgG	1978359	22	88412828	0.02238	717	16.04386	1.37124
TgT	1641895	8	88412828	0.01857	717	13.31525	0.60081
gAA	1691660	34	88412828	0.01913	717	13.71883	2.47835
gAC	1165686	12	88412828	0.01318	717	9.45334	1.26939
gAG	1825637	23	88412828	0.02065	717	14.80534	1.55349
gAT	1121271	16	88412828	0.01268	717	9.09315	1.75957
gCA	1572329	23	88412828	0.01778	717	12.75109	1.80377
gCC	1540454	22	88412828	0.01742	717	12.49259	1.76104
gCG	563085	4	88412828	0.00637	717	4.56644	0.87596
gCT	1673047	35	88412828	0.01892	717	13.56788	2.57962
gGA	1741070	18	88412828	0.01969	717	14.11953	1.27483
gGC	1540454	8	88412828	0.01742	717	12.49259	0.64038
gGG	1572721	17	88412828	0.01779	717	12.75427	1.33289
gGT	1314059	9	88412828	0.01486	717	10.65660	0.84455
gTA	875867	4	88412828	0.00991	717	7.10300	0.56314
gTC	1165686	3	88412828	0.01318	717	9.45334	0.31735
gTG	1564626	4	88412828	0.01770	717	12.68862	0.31524
gTT	1093390	7	88412828	0.01237	717	8.86705	0.78944

Calculation process of site preference ratio described in Methods was presented.

Supplementary figure legends

Supplementary Figure S1. 8-oxoG induced G to T transversion mutations in germ lineage cells. Germline mutations are defined as inheritable mutations that exist in the genome of terminally differentiated gametes that did not exist in the genome of the original fertilized egg. They are generated during development from a fertilized egg to terminally differentiated gametes (indicated in red). The reference genome sequence of an individual is defined as the sequence of the original fertilized egg, which is experimentally represented by the sequence of a somatic cell population, such as tail DNA. It is important to recognize that germline mutations mostly occur during mitoses, because germ lineage cells from fertilized eggs to differentiated sperm and eggs require large numbers of mitoses and only one meiosis.

A spontaneous mutation is defined as a mutation caused by cell endogenous events. ROS are produced by both cell endogenous events, such as mitochondrial activities, and by exogenous factors, such as irradiation and exposure to chemicals. All TOY-KO mice were maintained in a well-controlled room without any treatments; therefore, the mutations observed in the TOY-KO mouse can be considered as spontaneous mutations that could not be prevented in the absence of OGG1, MTH1, and MUTYH. MTH1 is an oxidized purine nucleotide triphosphatase to degrade 8-oxodGTP in the nucleotide pool. OGG1 is a DNA glycosylase that removes 8-oxoG from DNA. MUTYH is a DNA glycosylase that removes mis-incorporated A nucleotides opposite 8-oxoG in DNA. BER: Base Excision Repair.

Supplementary Figure S2. Hereditary traits observed in the TOY-KO mice pedigree.
(a) Pedigree of TOY-KO mice. The first TOY-KO mice (G0), 32M and 44F, were obtained by mating of $Ogg1^{-/-}/Mth1^{+/-}/Mutyh^{+/-}$ mice obtained by mating a C57BL/6J background (N>16) $Ogg1^{+/-}$, $Mth1^{+/-}$, and $Mutyh^{+/-}$ mice. Blue, yellow, orange and double underlines indicate mice with hydrocephalus, belly white spot, microphthalmia or anophthalmia and any

tumors distinguishable by macroscopic observation, respectively. Forty-two out of 117 TOY-KO mice possessed a tumor, such as a Harderian gland, skin, breast, ovarian tumors or lymphoma. (b) Belly white spot. Pedigrees of the TOY-KO mouse with belly white spot mated with C57BL/6J mice (shown as B6) are shown in the right. Yellow indicates mice with belly white spot. (c) Anophthalmia (left) and microphthalmia (right).

Supplementary Figure S3. Sequence signals are not detected in *Mutyh, Mth1,* and *Ogg1* targeted alleles. (a) Genome structures of the targeted loci of *Mth1, Ogg1*, and *Mutyh* genes[13,14,16]. (b) No sequencing reads corresponding to *Mutyh, Mth1*, and *Ogg1* targeted alleles were obtained in chromosomes 4, 5, and 6 of three TOY-KO mice, respectively. A DBF1 mouse was used as the wild-type control, which possesses intact *Mutyh, Mth1* and *Ogg1* genes.

Supplementary Figure S4. Mutation mosaicism in tail tissue. (a) Mutations #257, #261 and #262 (boxed by triangles) were initially found in males with a heterozygous status. Boxed red circles show the theoretical manner of transmission of the mutation to the offspring. The table was extracted from Supplementary Data S1. A G to T mutation is presented as G>T or C>A (opposite strand). (b) Expected mutation allele ratio for the X chromosome. In the case of males, either 100% A or 100% C is expected. (c)-(e) Mutation of the male X chromosome in a cell during early embryonic development resulted in a partially heterozygous genotype caused by mosaicism in the tail tissue. MassArray sequence data of mutations #257, #261 and #262 are shown in (c), (d) and (e), respectively. 135M (#257 and #262) or 87M (#261), a mutation origin mice, showed a C/A mixed signals.

Supplementary Figure S5. Previous model of 8-oxoG-induced mutations in mammals. Analogous to the *E. coli* system[18], three enzymes are considered to prevent 8-oxoG-dependent mutations. MTH1 (*mutT* homologue 1, NUDT1) degrades 8-oxodGTP in the nucleotide pool to prevent incorporation of the oxidized nucleotide into DNA[9]. OGG1 (8-oxoG DNA glycosylase) excises 8-oxoG from DNA[10,11], and MUTYH (*mutY* homologue, adenine DNA glycosylase) removes misincorporated A nucleotides opposite 8-oxoG in DNA[12]. However, we detected no A to C germline mutations in TOY-KO mice. In contrast to the *E. coli* system, the A to C mutation is prevented by another system in mammalian germ lineage cells.

Supplementary Data S1. Details of *de novo* germline mutations detected in TOY-KO mice. Two hundred and sixty-two mutations were detected in TOY365, TOY450, and TOY609 (chromosomes 1–3, 7–19, and X). About 60% of these mutations included 139 non-synonymous coding mutations, 16 stop-gained mutations, and one stop-lost mutation, affecting the amino acid sequences of proteins. The mutation status of each locus of each TOY-KO mouse in the pedigree is indicated by color. Information provided includes mutation ID, mutation type, mutation status of each mouse, mutation origin mouse, chromosome, position, gene, transcript ID, consequence, amino acid change, and the surrounding sequence of each mutation locus.
